# Crystal structure of (6*E*,20*E*)-3,24-di­fluoro-13,14,28,29-tetra­hydro-5*H*,22*H*-tetra­benzo[*e*,*j*,*p*,*u*][1,4,12,15]tetra­oxa­cyclo­docosine-5,22-dione

**DOI:** 10.1107/S2056989016018934

**Published:** 2017-01-01

**Authors:** Shaaban K. Mohamed, Mehmet Akkurt, Farouq E. Hawaiz, Mzgin M Ayoob, Eric Hosten

**Affiliations:** a Chemistry and Environmental Division, Manchester Metropolitan University, Manchester, M1 5GD, England, and, Chemistry Department, Faculty of Science, Minia University, 61519 El-Minia, Egypt; b Department of Physics, Faculty of Sciences, Erciyes University, 38039 Kayseri, Turkey; c Chemistry Department, College of Education, Salahaddin University-Hawler, Erbil, Kurdistan Region, Iraq; d Chemistry Department, Summerstrand Campus (South), PO Box 77000, Nelson Mandela, Metropolitan University, South Africa

**Keywords:** crystal structure, cone-shaped conformation, hydrogen bonds, crown ether, supra­molecular compounds.

## Abstract

The conformation of the title compound, C_34_H_26_F_2_O_6_, is cone-shaped, partially determined by intra­molecular C—H⋯O short contacts. The benzene rings at the top of the cone are inclined to one another by 73.10 (7)°, while the benzene rings at the bottom of the cone are inclined to one another by 35.49 (8)°. In the crystal, mol­ecules are linked by C—H⋯O and C—H⋯F hydrogen bonds, forming a three-dimensional supra­molecular structure. There are also C—H⋯π contacts present within the framework structure.

## Abstract 2

The conformation of the title compound is cone-shaped, partially determined by short intra­molecular C—H⋯O contacts. In the crystal, mol­ecules are linked *via* C—H⋯O and C—H⋯F hydrogen bonds and C—H⋯π inter­actions, forming a three-dimensional supra­molecular structure.

## Chemical context   

Macrocyclic compounds are known for their various applications, particularly in coordination chemistry (Delgado; 1995 ▸). The study of synthetic macrocyclic compounds is an important area of chemistry in view of their presence in many biologically significant naturally occurring metal complexes. Such compounds have received special attention because of their presence in many important biological systems such as metallo-porphyrins (for example haemoglobin, myoglobin, cytochromes, chloro­phylls), corrins (vitamin B12) and anti­biotics (valinomycin, nona­ctin) with anti­biotic, anti­fungal, anti­cancer and immunosuppressive activities as seen for erythromycin (McGuire *et al.*, 1952 ▸; Woodward *et al.*; 1981 ▸), amphotericin B (Vandeputte *et al.*, 1956 ▸; Nicolaou *et al.*, 1988 ▸), epithilone B (Gerth *et al.*, 1996 ▸; Bode & Carreira; 2001 ▸) and rapamycin (Vezina *et al.*, 1975 ▸; Smith *et al.*, 1997 ▸). In addition, macrocyclic compounds having ether linkages and chalcone moieties have important applications (Rina *et al.*, 2012 ▸, Matsushima *et al.*, 2001 ▸). In this context the title compound was prepared and herein we report on its synthesis and crystal structure.

## Structural commentary   

**Figure 1:**
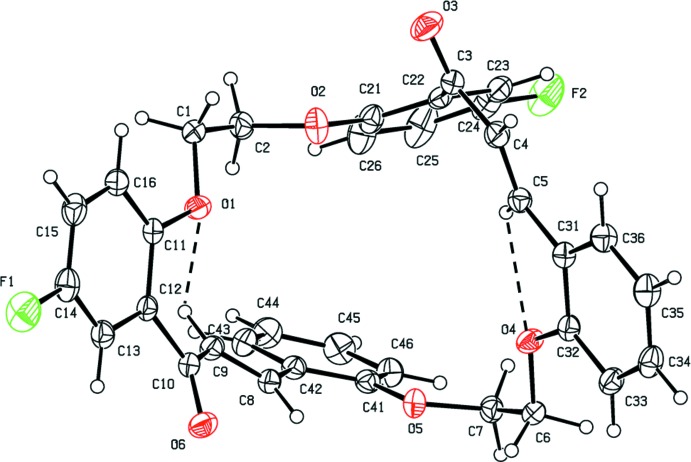
A view of the mol­ecular structure of the title compound, with atom labelling and 50% probability displacement ellipsoids. The short intra­molecular C—H⋯O contacts are shown as dashed lines (see Table 1 ▸).

**Figure 2:**
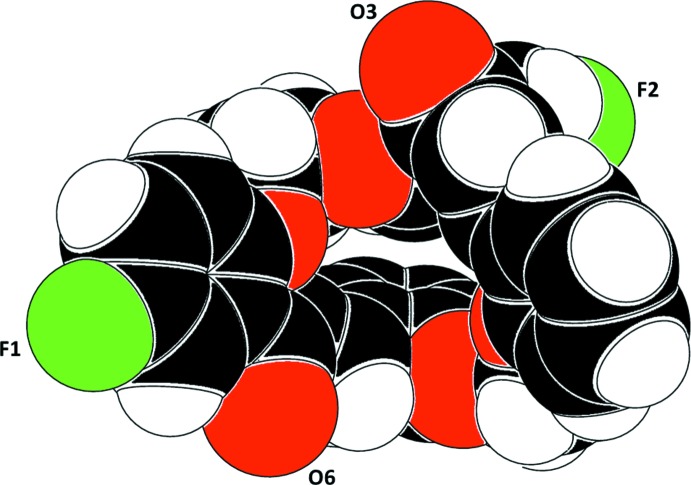
A CPK model of the title compound, illustrating the cone-shaped conformation.

The title compound, Fig. 1 ▸, has a cone-shaped conformation, partially determined by intra­molecular C—H⋯O short contacts (Table 1 ▸ and Fig. 1 ▸). The benzene rings at the top of the cone (C11–C16 and C31–C36) are inclined to one another by 73.10 (7)°, while the benzene rings at the bottom of the cone (C21–C26 and C41–C46) are inclined to one another by 35.49 (8)° (Fig. 2 ▸). The bond lengths and angles are similar to those observed in one of the starting materials for the synthesis of the title compound, *viz.* 2,2′-[ethane-1,2-diylbis(­oxy)]dibenzaldehyde (Aravindan *et al.*, 2003 ▸; Zhang *et al.*, 2003 ▸); both measured at room temperature. A low temperature (120 K) structure analysis of the same compound has also been reported (Akkurt *et al.*, 2013 ▸).
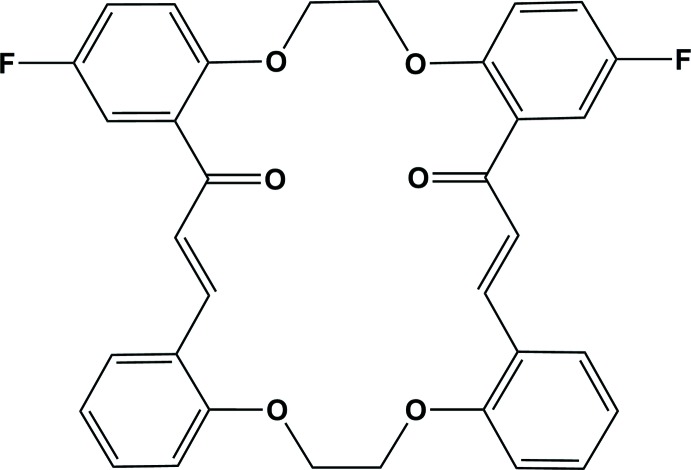


## Supra­molecular features   

**Figure 3:**
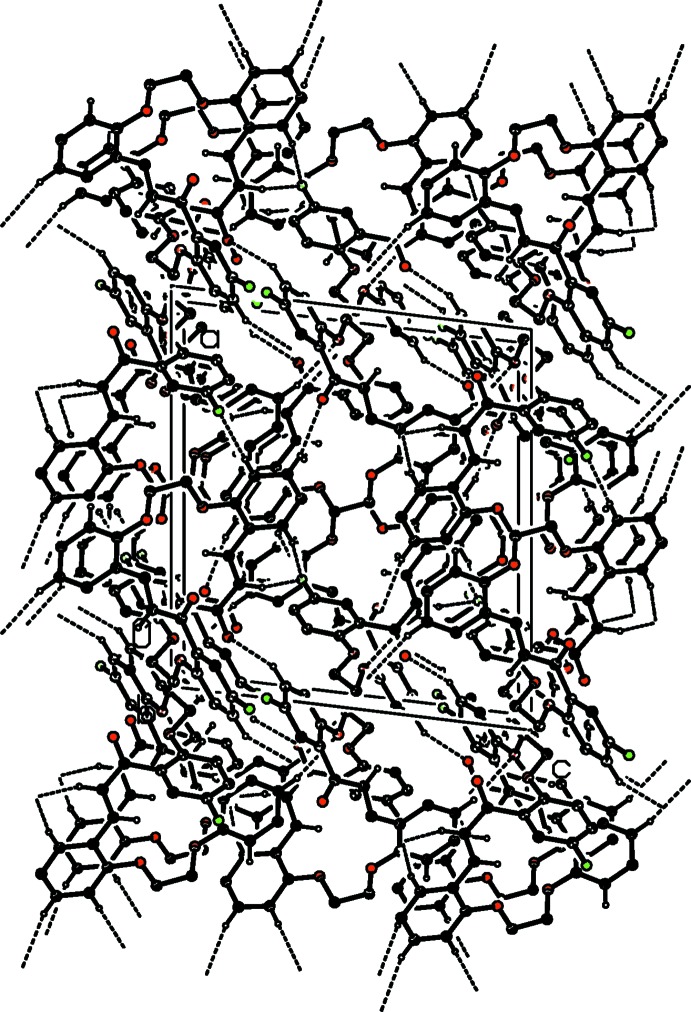
The crystal packing of the title compound, viewed along the *b* axis. Hydrogen bonds are shown as dashed lines (see Table 1 ▸), and for clarity only the H atoms involved in hydrogen bonding have been included.

In the crystal, mol­ecules are linked by C—H⋯O and C—H⋯F hydrogen bonds, forming a three-dimensional supra­molecular structure (Fig. 3 ▸ and Table 1 ▸). There are also C—H⋯π inter­actions present, involving inversion-related mol­ecules, within the three-dimensional framework (Table 1 ▸).

## Database survey   

A search of the Cambridge Structural Database (Version 5.37, update May 2016; Groom *et al.*, 2016 ▸) indicated the presence of the subunit 1,2-bis­(2-vinyl­phen­oxy)ethane in a number of macrocyclic-type compounds. However, no macrocyclic-type compounds were found containing the subunit 2,2′-[ethane-1,2-diylbis(­oxy)]dibenzaldehyde. The title compound, which contains both these subunits, is unique; no other reports of mol­ecules of this type were found.

## Synthesis and crystallization   

**Figure 4:**
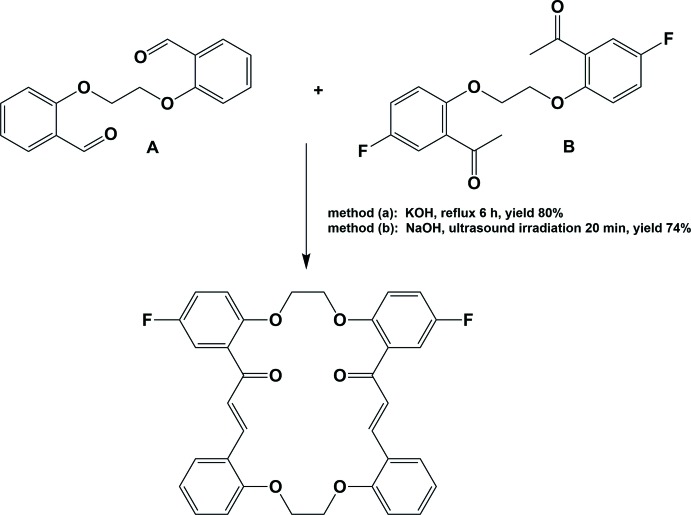
Reaction scheme.

The title compound has been synthesized by two methods, illustrated in Fig. 4 ▸.

**Method (** ***a*** **): High-dilution method**

A mixture of 2,2′-[ethane-1,2-diylbis(­oxy)]dibenzaldehyde (**A**) (67.6 mg; 0.25 mmol) and 1,1′-{(ethane-1,2-diylbis(­oxy)]bis­(5-fluoro-2,1-phenyl­ene)}bis­(ethan-1-one) (**B**) (83.6 mg; 0.25 mmol) was dissolved in a KOH solution (10%, 130–160 ml) in MeOH/H_2_O (3:1) and the mixture was refluxed for 6 h. The reaction mixture was left at room temperature with stirring for *ca* four days, then the solvent was reduced to nearly half volume under reduced pressure. The resulting precipitate was collected by filtration, dried and recrystallized from chloro­form/methanol solution (1:1) to give yellow block-shaped crystals, suitable for x-ray diffraction (yield 80%, m.p. 553–554 K).

**Method (** ***b*** **): Ultrasound-assisted synthesis**

Compound **A** (0.55 mmol, 0.15 gm) was dissolved in ethanol (5 ml) and added to a solution of compound **B** (0.55 mmol) in ethanol (5 ml), and solid NaOH (0.3 gm) was added to the mixture. The mixture was then irradiated in the water bath of an ultrasonic cleaner at room temperature for 20 min. The mixture solidified and the product was separated by filtration under vacuum, washed with ethanol, dried and purified by recrystallization from chloro­form solution (yield 74%). Single crystals were obtained by slow evaporation of a dilute solution of the title compound in chloro­form over 13 days at room temperature (m.p. 553–554 K).

## Refinement   

Crystal data, data collection and structure refinement details are summarized in Table 2 ▸. All H atoms were positioned geometrically and refined using a riding model: C—H = 0.95–0.99 Å with *U*_iso_(H) = 1.2*U*_eq_(C).

## Supplementary Material

Crystal structure: contains datablock(s) I, Global. DOI: 10.1107/S2056989016018934/su5336sup1.cif

Structure factors: contains datablock(s) I. DOI: 10.1107/S2056989016018934/su5336Isup2.hkl

CCDC reference: 1519443

Additional supporting information: crystallographic information; 3D view; checkCIF report

## Figures and Tables

**Table 1:** Hydrogen-bond geometry (Å, °) *Cg*3 is the centroid of the C31–C36 ring.

*D*—H⋯*A*	*D*—H	H⋯*A*	*D*⋯*A*	*D*—H⋯*A*
C5—H5⋯O4	0.95	2.35	2.7023 (16)	101
C9—H9⋯O1	0.95	2.40	2.7281 (16)	100
C4—H4⋯F2^i^	0.95	2.37	3.1387 (17)	138
C15—H15⋯O3^ii^	0.95	2.51	3.3211 (19)	143
C33—H33⋯F2^iii^	0.95	2.53	3.483 (2)	176
C34—H34⋯O6^iv^	0.95	2.51	3.3649 (17)	150
C36—H36⋯F2^i^	0.95	2.43	3.3380 (19)	161
C44—H44⋯O1^v^	0.95	2.58	3.4986 (18)	163
C46—H46⋯*Cg*3^iii^	0.95	2.84	3.6829 (16)	149

Symmetry codes: (i) 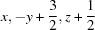; (ii) 
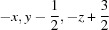
; (iii) 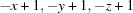; (iv) 
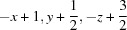
; (v) 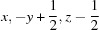.

**Table 2:** Experimental details

Crystal data	
Chemical formula	C_34_H_26_F_2_O_6_
*M*_r_	568.55
Crystal system, space group	Monoclinic, *P*2_1_/*c*
Temperature (K)	200
*a*, *b*, *c* (Å)	16.2618 (7), 11.6708 (5), 14.7359 (7)
β (°)	96.945 (2)
*V* (Å^3^)	2776.2 (2)
*Z*	4
Radiation type	Mo *K*α
μ (mm^−1^)	0.10
Crystal size (mm)	0.63 × 0.29 × 0.15

Data collection	
Diffractometer	Bruker APEXII CCD
Absorption correction	Multi-scan (*SADABS*; Bruker, 2009 ▸)
*T*_min_, *T*_max_	0.894, 1.000
No. of measured, independent and observed [*I* > 2σ(*I*)] reflections	38582, 6911, 5302
*R*_int_	0.020
(sin θ/λ)_max_ (Å^−1^)	0.668

Refinement	
*R*[*F* ^2^ > 2σ(*F* ^2^)], *wR*(*F* ^2^), *S*	0.040, 0.106, 1.01
No. of reflections	6911
No. of parameters	379
H-atom treatment	H-atom parameters constrained
Δρ_max_, Δρ_min_ (e Å^−3^)	0.32, −0.39

Computer programs: *APEX2* and *SAINT* (Bruker, 2009 ▸), *SHELXT2014/7* (Sheldrick, 2015*a* ▸), *SHELXL2014/7* (Sheldrick, 2015*b* ▸) and *PLATON* (Spek, 2009 ▸).

## supplementary crystallographic information

### Crystal data

**Table:**

C_34_H_26_F_2_O_6_	*F*(000) = 1184
*M**_r_* = 568.55	*D*_x_ = 1.360 Mg m^−^^3^
Monoclinic, *P*2_1_/*c*	Mo *K*α radiation, λ = 0.71073 Å
*a* = 16.2618 (7) Å	Cell parameters from 9878 reflections
*b* = 11.6708 (5) Å	θ = 2.5–28.2°
*c* = 14.7359 (7) Å	µ = 0.10 mm^−^^1^
β = 96.945 (2)°	*T* = 200 K
*V* = 2776.2 (2) Å^3^	Block, yellow
*Z* = 4	0.63 × 0.29 × 0.15 mm

### Data collection

**Table:**

Bruker APEXII CCD diffractometer	6911 independent reflections
Radiation source: sealed tube	5302 reflections with *I* > 2σ(*I*)
Graphite monochromator	*R*_int_ = 0.020
Detector resolution: 8.3333 pixels mm^-1^	θ_max_ = 28.3°, θ_min_ = 2.5°
φ and ω scans	*h* = −21→20
Absorption correction: multi-scan (SADABS; Bruker, 2009)	*k* = −15→15
*T*_min_ = 0.894, *T*_max_ = 1.000	*l* = −19→19
38582 measured reflections	

### Refinement

**Table:**

Refinement on *F*^2^	Primary atom site location: structure-invariant direct methods
Least-squares matrix: full	Secondary atom site location: difference Fourier map
*R*[*F*^2^ > 2σ(*F*^2^)] = 0.040	Hydrogen site location: inferred from neighbouring sites
*wR*(*F*^2^) = 0.106	H-atom parameters constrained
*S* = 1.01	*w* = 1/[σ^2^(*F*_o_^2^) + (0.0392*P*)^2^ + 1.1029*P*] where *P* = (*F*_o_^2^ + 2*F*_c_^2^)/3
6911 reflections	(Δ/σ)_max_ < 0.001
379 parameters	Δρ_max_ = 0.32 e Å^−^^3^
0 restraints	Δρ_min_ = −0.39 e Å^−^^3^

### Special details

**Table:**

Geometry. All esds (except the esd in the dihedral angle between two l.s. planes) are estimated using the full covariance matrix. The cell esds are taken into account individually in the estimation of esds in distances, angles and torsion angles; correlations between esds in cell parameters are only used when they are defined by crystal symmetry. An approximate (isotropic) treatment of cell esds is used for estimating esds involving l.s. planes.

### Fractional atomic coordinates and isotropic or equivalent isotropic displacement parameters (Å^2^)

**Table:**

	*x*	*y*	*z*	*U*_iso_*/*U*_eq_	
F1	−0.00518 (7)	−0.16078 (10)	0.73153 (8)	0.0712 (3)	
F2	0.30286 (7)	0.77217 (11)	0.36051 (9)	0.0823 (4)	
O1	0.07789 (6)	0.21042 (8)	0.53657 (8)	0.0426 (2)	
O2	0.12188 (7)	0.44266 (9)	0.50741 (7)	0.0479 (3)	
O3	0.13697 (6)	0.63181 (11)	0.65378 (8)	0.0567 (3)	
O4	0.45695 (6)	0.39443 (9)	0.59069 (6)	0.0389 (2)	
O5	0.41025 (6)	0.24383 (8)	0.44309 (6)	0.0365 (2)	
O6	0.23814 (6)	−0.05205 (9)	0.57921 (8)	0.0473 (3)	
C1	0.02059 (8)	0.30158 (12)	0.51429 (11)	0.0423 (3)	
H1A	0.0096	0.3429	0.5703	0.051*	
H1B	−0.0325	0.2714	0.4835	0.051*	
C2	0.05993 (9)	0.37973 (13)	0.45186 (11)	0.0433 (3)	
H2A	0.0851	0.3349	0.4053	0.052*	
H2B	0.0181	0.4324	0.4202	0.052*	
C3	0.20104 (8)	0.60305 (12)	0.62506 (10)	0.0376 (3)	
C4	0.27425 (8)	0.57029 (12)	0.68694 (10)	0.0388 (3)	
H4	0.2730	0.5805	0.7507	0.047*	
C5	0.34280 (8)	0.52688 (11)	0.65906 (9)	0.0335 (3)	
H5	0.3429	0.5177	0.5950	0.040*	
C6	0.51460 (8)	0.32791 (12)	0.54755 (10)	0.0368 (3)	
H6A	0.5213	0.2511	0.5760	0.044*	
H6B	0.5693	0.3661	0.5535	0.044*	
C7	0.48031 (8)	0.31774 (12)	0.44922 (9)	0.0372 (3)	
H7A	0.4638	0.3942	0.4242	0.045*	
H7B	0.5228	0.2859	0.4135	0.045*	
C8	0.26989 (8)	0.11337 (11)	0.44357 (9)	0.0337 (3)	
H8	0.3169	0.0888	0.4834	0.040*	
C9	0.19546 (8)	0.08387 (12)	0.46483 (9)	0.0352 (3)	
H9	0.1477	0.1089	0.4264	0.042*	
C10	0.18427 (8)	0.01401 (11)	0.54551 (9)	0.0336 (3)	
C11	0.05441 (8)	0.11931 (11)	0.58468 (9)	0.0341 (3)	
C12	0.10509 (8)	0.02187 (11)	0.58816 (9)	0.0321 (3)	
C13	0.08374 (9)	−0.07270 (12)	0.63846 (10)	0.0387 (3)	
H13	0.1166	−0.1402	0.6413	0.046*	
C14	0.01517 (10)	−0.06726 (14)	0.68353 (10)	0.0454 (4)	
C15	−0.03344 (9)	0.02861 (14)	0.68325 (11)	0.0468 (4)	
H15	−0.0795	0.0305	0.7173	0.056*	
C16	−0.01442 (9)	0.12229 (13)	0.63273 (10)	0.0421 (3)	
H16	−0.0482	0.1889	0.6306	0.050*	
C21	0.16576 (9)	0.52274 (13)	0.46597 (10)	0.0399 (3)	
C22	0.20682 (8)	0.60381 (12)	0.52387 (10)	0.0352 (3)	
C23	0.25285 (8)	0.68864 (13)	0.48752 (12)	0.0452 (4)	
H23	0.2808	0.7455	0.5257	0.054*	
C24	0.25707 (9)	0.68851 (16)	0.39503 (13)	0.0559 (5)	
C25	0.21969 (11)	0.6085 (2)	0.33750 (12)	0.0684 (6)	
H25	0.2256	0.6099	0.2742	0.082*	
C26	0.17280 (11)	0.52466 (18)	0.37308 (11)	0.0607 (5)	
H26	0.1454	0.4683	0.3339	0.073*	
C31	0.41776 (8)	0.49208 (11)	0.71727 (9)	0.0329 (3)	
C32	0.47646 (8)	0.42363 (11)	0.68041 (9)	0.0332 (3)	
C33	0.54911 (8)	0.39044 (13)	0.73364 (10)	0.0393 (3)	
H33	0.5881	0.3429	0.7085	0.047*	
C34	0.56427 (9)	0.42722 (14)	0.82355 (10)	0.0442 (3)	
H34	0.6142	0.4056	0.8597	0.053*	
C35	0.50782 (10)	0.49464 (14)	0.86091 (10)	0.0455 (4)	
H35	0.5188	0.5196	0.9225	0.055*	
C36	0.43489 (9)	0.52605 (13)	0.80844 (10)	0.0409 (3)	
H36	0.3957	0.5716	0.8350	0.049*	
C41	0.35808 (8)	0.24427 (11)	0.36284 (9)	0.0333 (3)	
C42	0.28472 (8)	0.18095 (11)	0.36335 (9)	0.0337 (3)	
C43	0.22821 (9)	0.18261 (13)	0.28370 (10)	0.0429 (3)	
H43	0.1776	0.1418	0.2828	0.051*	
C44	0.24391 (10)	0.24171 (15)	0.20655 (11)	0.0503 (4)	
H44	0.2045	0.2416	0.1534	0.060*	
C45	0.31735 (11)	0.30101 (14)	0.20740 (11)	0.0501 (4)	
H45	0.3289	0.3408	0.1542	0.060*	
C46	0.37440 (9)	0.30306 (13)	0.28523 (10)	0.0419 (3)	
H46	0.4246	0.3447	0.2854	0.050*	

### Atomic displacement parameters (Å^2^)

**Table:**

	*U*^11^	*U*^22^	*U*^33^	*U*^12^	*U*^13^	*U*^23^
F1	0.0696 (7)	0.0713 (7)	0.0754 (7)	−0.0146 (6)	0.0193 (6)	0.0290 (6)
F2	0.0549 (6)	0.0891 (8)	0.1024 (9)	−0.0128 (6)	0.0076 (6)	0.0586 (7)
O1	0.0308 (5)	0.0332 (5)	0.0649 (7)	0.0023 (4)	0.0108 (4)	0.0063 (5)
O2	0.0516 (6)	0.0491 (6)	0.0411 (6)	−0.0208 (5)	−0.0019 (5)	0.0028 (5)
O3	0.0340 (5)	0.0756 (8)	0.0623 (7)	0.0065 (5)	0.0129 (5)	−0.0085 (6)
O4	0.0311 (5)	0.0489 (6)	0.0351 (5)	0.0073 (4)	−0.0020 (4)	−0.0084 (4)
O5	0.0345 (5)	0.0411 (5)	0.0330 (5)	−0.0087 (4)	0.0010 (4)	−0.0006 (4)
O6	0.0344 (5)	0.0437 (6)	0.0630 (7)	0.0062 (4)	0.0029 (5)	0.0083 (5)
C1	0.0292 (6)	0.0323 (7)	0.0637 (10)	0.0012 (5)	−0.0018 (6)	−0.0020 (6)
C2	0.0398 (7)	0.0360 (7)	0.0506 (8)	−0.0017 (6)	−0.0087 (6)	−0.0006 (6)
C3	0.0306 (6)	0.0341 (7)	0.0485 (8)	−0.0021 (5)	0.0063 (6)	−0.0064 (6)
C4	0.0370 (7)	0.0429 (8)	0.0363 (7)	−0.0010 (6)	0.0031 (5)	−0.0087 (6)
C5	0.0341 (6)	0.0335 (7)	0.0324 (6)	−0.0010 (5)	0.0019 (5)	−0.0022 (5)
C6	0.0268 (6)	0.0379 (7)	0.0453 (8)	0.0013 (5)	0.0021 (5)	−0.0061 (6)
C7	0.0329 (6)	0.0390 (7)	0.0408 (7)	−0.0060 (6)	0.0087 (5)	−0.0057 (6)
C8	0.0298 (6)	0.0342 (7)	0.0361 (7)	−0.0005 (5)	0.0003 (5)	−0.0046 (5)
C9	0.0303 (6)	0.0383 (7)	0.0364 (7)	0.0015 (5)	0.0009 (5)	−0.0041 (6)
C10	0.0275 (6)	0.0322 (6)	0.0401 (7)	−0.0028 (5)	−0.0008 (5)	−0.0046 (5)
C11	0.0288 (6)	0.0327 (6)	0.0401 (7)	−0.0067 (5)	0.0013 (5)	−0.0045 (5)
C12	0.0274 (6)	0.0345 (7)	0.0331 (6)	−0.0046 (5)	−0.0013 (5)	−0.0036 (5)
C13	0.0373 (7)	0.0374 (7)	0.0393 (7)	−0.0058 (6)	−0.0032 (6)	0.0012 (6)
C14	0.0448 (8)	0.0500 (9)	0.0410 (8)	−0.0150 (7)	0.0034 (6)	0.0065 (7)
C15	0.0398 (8)	0.0572 (9)	0.0453 (8)	−0.0138 (7)	0.0129 (6)	−0.0087 (7)
C16	0.0338 (7)	0.0408 (8)	0.0524 (9)	−0.0051 (6)	0.0086 (6)	−0.0122 (6)
C21	0.0343 (7)	0.0443 (8)	0.0395 (7)	−0.0034 (6)	−0.0021 (6)	0.0082 (6)
C22	0.0248 (6)	0.0350 (7)	0.0451 (7)	0.0041 (5)	0.0016 (5)	0.0059 (6)
C23	0.0299 (7)	0.0387 (8)	0.0661 (10)	0.0003 (6)	0.0020 (6)	0.0107 (7)
C24	0.0336 (7)	0.0656 (11)	0.0672 (11)	−0.0028 (7)	0.0011 (7)	0.0377 (9)
C25	0.0500 (10)	0.1086 (16)	0.0438 (9)	−0.0169 (10)	−0.0062 (7)	0.0300 (10)
C26	0.0565 (10)	0.0842 (13)	0.0384 (8)	−0.0195 (9)	−0.0065 (7)	0.0085 (8)
C31	0.0329 (6)	0.0339 (6)	0.0314 (6)	−0.0037 (5)	0.0021 (5)	0.0016 (5)
C32	0.0317 (6)	0.0343 (7)	0.0326 (6)	−0.0045 (5)	−0.0002 (5)	0.0012 (5)
C33	0.0315 (6)	0.0422 (8)	0.0426 (8)	−0.0012 (6)	−0.0016 (5)	0.0035 (6)
C34	0.0383 (7)	0.0506 (9)	0.0405 (8)	−0.0078 (6)	−0.0084 (6)	0.0112 (7)
C35	0.0506 (8)	0.0539 (9)	0.0304 (7)	−0.0085 (7)	−0.0025 (6)	0.0022 (6)
C36	0.0441 (8)	0.0448 (8)	0.0337 (7)	−0.0022 (6)	0.0038 (6)	−0.0022 (6)
C41	0.0344 (6)	0.0337 (6)	0.0315 (6)	0.0026 (5)	0.0029 (5)	−0.0031 (5)
C42	0.0332 (6)	0.0338 (7)	0.0340 (7)	0.0021 (5)	0.0033 (5)	−0.0037 (5)
C43	0.0355 (7)	0.0469 (8)	0.0442 (8)	−0.0007 (6)	−0.0036 (6)	−0.0021 (6)
C44	0.0521 (9)	0.0539 (9)	0.0411 (8)	0.0043 (8)	−0.0105 (7)	0.0036 (7)
C45	0.0602 (10)	0.0506 (9)	0.0383 (8)	0.0006 (8)	0.0010 (7)	0.0085 (7)
C46	0.0438 (8)	0.0429 (8)	0.0392 (7)	−0.0038 (6)	0.0055 (6)	0.0021 (6)

### Geometric parameters (Å, º)

**Table:**

F1—C14	1.3628 (18)	C12—C13	1.3966 (19)
F2—C24	1.3632 (18)	C13—C14	1.367 (2)
O1—C11	1.3581 (16)	C13—H13	0.9500
O1—C1	1.4262 (16)	C14—C15	1.370 (2)
O2—C21	1.3643 (17)	C15—C16	1.379 (2)
O2—C2	1.4230 (17)	C15—H15	0.9500
O3—C3	1.2183 (16)	C16—H16	0.9500
O4—C32	1.3651 (16)	C21—C26	1.388 (2)
O4—C6	1.4247 (16)	C21—C22	1.390 (2)
O5—C41	1.3695 (15)	C22—C23	1.387 (2)
O5—C7	1.4231 (15)	C23—C24	1.373 (2)
O6—C10	1.2261 (16)	C23—H23	0.9500
C1—C2	1.493 (2)	C24—C25	1.355 (3)
C1—H1A	0.9900	C25—C26	1.382 (3)
C1—H1B	0.9900	C25—H25	0.9500
C2—H2A	0.9900	C26—H26	0.9500
C2—H2B	0.9900	C31—C36	1.3962 (19)
C3—C4	1.460 (2)	C31—C32	1.4035 (19)
C3—C22	1.505 (2)	C32—C33	1.3918 (18)
C4—C5	1.3338 (19)	C33—C34	1.386 (2)
C4—H4	0.9500	C33—H33	0.9500
C5—C31	1.4608 (18)	C34—C35	1.374 (2)
C5—H5	0.9500	C34—H34	0.9500
C6—C7	1.4931 (19)	C35—C36	1.385 (2)
C6—H6A	0.9900	C35—H35	0.9500
C6—H6B	0.9900	C36—H36	0.9500
C7—H7A	0.9900	C41—C46	1.3867 (19)
C7—H7B	0.9900	C41—C42	1.4041 (18)
C8—C9	1.3317 (18)	C42—C43	1.4006 (19)
C8—C42	1.4652 (19)	C43—C44	1.380 (2)
C8—H8	0.9500	C43—H43	0.9500
C9—C10	1.471 (2)	C44—C45	1.379 (2)
C9—H9	0.9500	C44—H44	0.9500
C10—C12	1.5022 (18)	C45—C46	1.385 (2)
C11—C16	1.3955 (19)	C45—H45	0.9500
C11—C12	1.4017 (19)	C46—H46	0.9500
			
C11—O1—C1	119.15 (11)	C14—C15—H15	120.6
C21—O2—C2	117.92 (11)	C16—C15—H15	120.6
C32—O4—C6	118.67 (10)	C15—C16—C11	120.02 (14)
C41—O5—C7	117.40 (10)	C15—C16—H16	120.0
O1—C1—C2	106.30 (11)	C11—C16—H16	120.0
O1—C1—H1A	110.5	O2—C21—C26	124.24 (14)
C2—C1—H1A	110.5	O2—C21—C22	115.48 (12)
O1—C1—H1B	110.5	C26—C21—C22	120.25 (14)
C2—C1—H1B	110.5	C23—C22—C21	119.38 (14)
H1A—C1—H1B	108.7	C23—C22—C3	119.20 (13)
O2—C2—C1	106.68 (12)	C21—C22—C3	121.41 (12)
O2—C2—H2A	110.4	C24—C23—C22	118.46 (15)
C1—C2—H2A	110.4	C24—C23—H23	120.8
O2—C2—H2B	110.4	C22—C23—H23	120.8
C1—C2—H2B	110.4	C25—C24—F2	118.96 (17)
H2A—C2—H2B	108.6	C25—C24—C23	123.32 (15)
O3—C3—C4	121.47 (14)	F2—C24—C23	117.70 (17)
O3—C3—C22	120.06 (13)	C24—C25—C26	118.48 (17)
C4—C3—C22	118.44 (12)	C24—C25—H25	120.8
C5—C4—C3	123.76 (13)	C26—C25—H25	120.8
C5—C4—H4	118.1	C25—C26—C21	120.06 (17)
C3—C4—H4	118.1	C25—C26—H26	120.0
C4—C5—C31	126.44 (13)	C21—C26—H26	120.0
C4—C5—H5	116.8	C36—C31—C32	117.91 (12)
C31—C5—H5	116.8	C36—C31—C5	122.77 (13)
O4—C6—C7	106.72 (10)	C32—C31—C5	119.30 (12)
O4—C6—H6A	110.4	O4—C32—C33	123.95 (12)
C7—C6—H6A	110.4	O4—C32—C31	115.31 (11)
O4—C6—H6B	110.4	C33—C32—C31	120.74 (12)
C7—C6—H6B	110.4	C34—C33—C32	119.55 (14)
H6A—C6—H6B	108.6	C34—C33—H33	120.2
O5—C7—C6	108.18 (11)	C32—C33—H33	120.2
O5—C7—H7A	110.1	C35—C34—C33	120.69 (13)
C6—C7—H7A	110.1	C35—C34—H34	119.7
O5—C7—H7B	110.1	C33—C34—H34	119.7
C6—C7—H7B	110.1	C34—C35—C36	119.78 (14)
H7A—C7—H7B	108.4	C34—C35—H35	120.1
C9—C8—C42	124.89 (12)	C36—C35—H35	120.1
C9—C8—H8	117.6	C35—C36—C31	121.33 (14)
C42—C8—H8	117.6	C35—C36—H36	119.3
C8—C9—C10	122.56 (12)	C31—C36—H36	119.3
C8—C9—H9	118.7	O5—C41—C46	123.62 (12)
C10—C9—H9	118.7	O5—C41—C42	115.56 (11)
O6—C10—C9	121.58 (12)	C46—C41—C42	120.82 (12)
O6—C10—C12	118.40 (12)	C43—C42—C41	117.39 (13)
C9—C10—C12	120.02 (11)	C43—C42—C8	121.89 (12)
O1—C11—C16	122.60 (13)	C41—C42—C8	120.70 (12)
O1—C11—C12	116.94 (11)	C44—C43—C42	121.96 (14)
C16—C11—C12	120.39 (13)	C44—C43—H43	119.0
C13—C12—C11	118.54 (12)	C42—C43—H43	119.0
C13—C12—C10	117.03 (12)	C45—C44—C43	119.33 (14)
C11—C12—C10	124.34 (12)	C45—C44—H44	120.3
C14—C13—C12	119.41 (14)	C43—C44—H44	120.3
C14—C13—H13	120.3	C44—C45—C46	120.58 (15)
C12—C13—H13	120.3	C44—C45—H45	119.7
F1—C14—C13	118.59 (15)	C46—C45—H45	119.7
F1—C14—C15	118.66 (14)	C45—C46—C41	119.89 (14)
C13—C14—C15	122.75 (14)	C45—C46—H46	120.1
C14—C15—C16	118.84 (14)	C41—C46—H46	120.1
			
C11—O1—C1—C2	−172.73 (12)	C4—C3—C22—C21	109.61 (15)
C21—O2—C2—C1	−178.45 (12)	C21—C22—C23—C24	−0.8 (2)
O1—C1—C2—O2	−75.81 (14)	C3—C22—C23—C24	−179.61 (13)
O3—C3—C4—C5	172.72 (15)	C22—C23—C24—C25	−1.2 (2)
C22—C3—C4—C5	−8.9 (2)	C22—C23—C24—F2	−179.71 (13)
C3—C4—C5—C31	−179.75 (13)	F2—C24—C25—C26	−179.42 (17)
C32—O4—C6—C7	−175.40 (11)	C23—C24—C25—C26	2.1 (3)
C41—O5—C7—C6	164.74 (11)	C24—C25—C26—C21	−0.9 (3)
O4—C6—C7—O5	−71.01 (14)	O2—C21—C26—C25	−179.07 (16)
C42—C8—C9—C10	178.86 (12)	C22—C21—C26—C25	−1.0 (3)
C8—C9—C10—O6	−25.3 (2)	C4—C5—C31—C36	−16.4 (2)
C8—C9—C10—C12	155.24 (13)	C4—C5—C31—C32	165.00 (14)
C1—O1—C11—C16	−16.33 (19)	C6—O4—C32—C33	−1.32 (19)
C1—O1—C11—C12	166.75 (12)	C6—O4—C32—C31	178.13 (12)
O1—C11—C12—C13	178.80 (11)	C36—C31—C32—O4	−179.10 (12)
C16—C11—C12—C13	1.80 (19)	C5—C31—C32—O4	−0.44 (18)
O1—C11—C12—C10	2.40 (19)	C36—C31—C32—C33	0.4 (2)
C16—C11—C12—C10	−174.60 (12)	C5—C31—C32—C33	179.02 (12)
O6—C10—C12—C13	−23.85 (18)	O4—C32—C33—C34	178.22 (13)
C9—C10—C12—C13	155.65 (12)	C31—C32—C33—C34	−1.2 (2)
O6—C10—C12—C11	152.60 (13)	C32—C33—C34—C35	0.9 (2)
C9—C10—C12—C11	−27.90 (18)	C33—C34—C35—C36	0.2 (2)
C11—C12—C13—C14	−0.82 (19)	C34—C35—C36—C31	−1.0 (2)
C10—C12—C13—C14	175.85 (12)	C32—C31—C36—C35	0.8 (2)
C12—C13—C14—F1	179.36 (13)	C5—C31—C36—C35	−177.86 (14)
C12—C13—C14—C15	−1.3 (2)	C7—O5—C41—C46	6.93 (19)
F1—C14—C15—C16	−178.23 (13)	C7—O5—C41—C42	−172.61 (11)
C13—C14—C15—C16	2.5 (2)	O5—C41—C42—C43	177.69 (12)
C14—C15—C16—C11	−1.4 (2)	C46—C41—C42—C43	−1.9 (2)
O1—C11—C16—C15	−177.51 (13)	O5—C41—C42—C8	−4.12 (18)
C12—C11—C16—C15	−0.7 (2)	C46—C41—C42—C8	176.32 (13)
C2—O2—C21—C26	−20.2 (2)	C9—C8—C42—C43	−26.3 (2)
C2—O2—C21—C22	161.70 (13)	C9—C8—C42—C41	155.60 (13)
O2—C21—C22—C23	−179.87 (12)	C41—C42—C43—C44	1.4 (2)
C26—C21—C22—C23	1.9 (2)	C8—C42—C43—C44	−176.79 (14)
O2—C21—C22—C3	−1.14 (19)	C42—C43—C44—C45	0.1 (2)
C26—C21—C22—C3	−179.34 (15)	C43—C44—C45—C46	−1.0 (3)
O3—C3—C22—C23	106.71 (16)	C44—C45—C46—C41	0.6 (2)
C4—C3—C22—C23	−71.66 (17)	O5—C41—C46—C45	−178.58 (14)
O3—C3—C22—C21	−72.03 (19)	C42—C41—C46—C45	0.9 (2)

### Hydrogen-bond geometry (Å, º)

Cg3 is the centroid of the C31–C36 ring.

**Table:**

*D*—H···*A*	*D*—H	H···*A*	*D*···*A*	*D*—H···*A*
C5—H5···O4	0.95	2.35	2.7023 (16)	101
C9—H9···O1	0.95	2.40	2.7281 (16)	100
C4—H4···F2^i^	0.95	2.37	3.1387 (17)	138
C15—H15···O3^ii^	0.95	2.51	3.3211 (19)	143
C33—H33···F2^iii^	0.95	2.53	3.483 (2)	176
C34—H34···O6^iv^	0.95	2.51	3.3649 (17)	150
C36—H36···F2^i^	0.95	2.43	3.3380 (19)	161
C44—H44···O1^v^	0.95	2.58	3.4986 (18)	163
C46—H46···*Cg*3^iii^	0.95	2.84	3.6829 (16)	149

Symmetry codes: (i) *x*, −*y*+3/2, *z*+1/2; (ii) −*x*, *y*−1/2, −*z*+3/2; (iii) −*x*+1, −*y*+1, −*z*+1; (iv) −*x*+1, *y*+1/2, −*z*+3/2; (v) *x*, −*y*+1/2, *z*−1/2.
